# The impact of a bundled policy intervention on improving the performance of rural healthcare in China

**DOI:** 10.1186/s12939-016-0334-8

**Published:** 2016-03-10

**Authors:** Jian Wu, Xiaofang Li, Yao Song, Hui Shao, Qian Shi, Doudou Qin, Shuangbao Xie, Lizheng Shi

**Affiliations:** College of Public Health, Zhengzhou University, Science Avenue 100, Gaoxin District, Zhengzhou, Henan Province 450001 PR China; College of Information and Engineering, Zhengzhou University, Zhengzhou, 450001 PR China; School of Public Health and Tropical Medicine, Tulane University, New Orleans, LA 70112 USA; Henan Provincial Health and Family Planning Committee, Zhengzhou, 450003 PR China

**Keywords:** Primary Health Care Services, Performance-related contracts, Policy evaluation, Rural health, China

## Abstract

**Background:**

The strategy of health policy has been changed for improving the performances to meeting the increasing healthcare demands. However, limited evidences were found to prove that the bundled payment was valid for service delivering in public sector. This study was designed to evaluate the effectiveness of a bundled policy on strengthening the county-village communication and improving the quality of chronic disease management.

**Methods:**

This is a retrospective cohort study using the data collected in 2011, 2012 and 2014 from the Rural Health Development Project in China. The policy intervention included performance-related contract with health facilities, developing technical guideline for doctors and nurses, routine monitoring of performance, and efforts to increase public awareness about the services. There were two intervention counties in Henan Province, China, while one county with similar characteristics in Henan was selected as control. Funding allocation, work load and salary for health care workers, volume of township-to-village technical assistance were reported before and after the policy was implemented. Our study also examined the policy impacts on improving treatment outcomes of diabetes and hypertension care.

**Results:**

There were substantial increases in the provision of the basic package of services including 96.6 % of patients with hypertension, 91.2 % of patients with diabetes under the health management system. After the intervention, there were 34.3 % (hypertension) and 42.0 % (diabetes) increase in regular follow-up visit rates, 24.6 and 17.2 % increase in blood pressure and blood glucose control rates, respectively. The family health records system covered 96 % of the rural families. Technical assistance between township health centres and village clinics were enhanced. Compared with baseline, the monthly training meeting and field supervision & guidance between township health centres and village clinics increased 1.0 meeting, 1.5 field visits, respectively, while the increases in the control county were only 0.3 meeting and 0.3 field visits. At the end of this study, 93.8 % of health workers achieved their performance goals. More patients were referred to appropriate levels of care.

**Conclusion:**

This bundled policy intervention effectively improved rural health care delivery. The result of our study can be used for local governments to implement performance-based health system management in developing country.

## Background

During the past two to three decades there has been a dramatic spread of health reform in many low and middle-income countries. In some cases this has been associated with a change in economic model and rapid economic growth; in other cases it has been associated with prolonged economic crisis, deterioration in the performance of the public sector and the increasing importance of informal markets. This phenomenon has stimulated interest in government strategies for influencing these markets to improve their performance in meeting health needs [1, 2]. One approach that has gained a lot of attention is the use of contracts linking payment of health service providers to their achievement of health care delivery targets [3–5]. However, very little evidence is available on in what circumstances delivery of services through contracts with the private sector is likely to be preferable to direct provision by the public sector [6, 7]. Despite enthusiasm about the potential for aligning financial incentives with high-quality health care, many fundamental questions about their optimal design, effectiveness, and mechanism of action remain unanswered [8]. Overall, the international record for pay for performance is growing and yet mixed in both OECD countries and transition economies [9].

This paper presents an intervention in Henan Province in China aimed at increasing access to the Basic Public Health Services (BPHS, similar to primary health care in US) [10, 11] through a combination of performance-related contracts with health facilities, routine monitoring of performance and efforts to increase public awareness about the services to which they are entitled. The payment mode which learns from the British experience for the pay-for-performance system: health workers earn points for each performance measure and payment is tied to the number of points received [12, 13]. Based on those experiences, the payment mode is built with adjustment to local policy environment. The facilities’ payment was defined by quarterly assessment and number of goals achieved listed in the contract. Individuals’ payment was defined by quantity and quality of works completed.

BPHS package includes the creation of health records for rural families, health education, and health care for children under three years old, maternal health care, health care for the elderly, immunization, reporting of infectious disease, management of hypertension and diabetes and treatment of severe mental illness. While the funding policy for this basic package of services are in place, the government faces a major challenge in ensuring that the additional funds reach local health facilities and are used to provide good quality health services [14].

The new BPHS policy required (and directly funded) clinicians to undertake population screening, especially for hypertension and diabetes, but also general health screening for the elderly, and covered both village level costs of screening and follow up, as well as confirmation on diagnosis and higher level care as needed at township hospitals. Patients pay in full for their drugs and other diagnostic tests and treatments, but this is largely reimbursed from the New Cooperative Rural Medical Scheme (NCRMS), the insurance system covering almost all rural citizens [15].

The aim of this study was to evaluate the effectiveness of the bundled policy including performance-related contract with health facilities, developing technical guideline for doctors and nurses, and routine monitoring of performance on strengthening the county-village communication and improving the quality of chronic disease management.

## Methods

### Design of the intervention

The Chinese Government established the China Rural Health Development Project (CRHDP, 2009–2014) [16], co-funded by the Government of China, a World Bank credit and a grant from the UK Department For International Development(DFID) to test BPHS practical strategies for implementing its rural health reform. Henan is one of eight participating provinces.

This study is a retrospective cohort study for evaluating the effect of BPHS intervention in Henan Province from 2011 to 2014. Each intervention county developed detailed management guidelines for each item in the package according to the National BPHS Guideline Book. These guidelines defined the roles of the township health centres and village clinics. The counties estimated the cost of delivering these services at township and village levels. They signed contracts with township health centres to deliver the package of services on the basis of these cost estimates and the township health centres signed sub-contracts with village doctors. These contracts defined the services to be provided, quality standards to be achieved, and methods of performance assessment and criteria for payment. Comparing to the previous payment system in which doctors and nurses received fixed salary regardless of their performance, this payment mode linked the amount of payment to the quantity and quality of individuals’ work. The funds were split more or less evenly between township and village levels. This was a major departure from the previous practice of allocating almost all government funding to government-owned township facilities.

Each county established a team to make quarterly monitoring visits to each township to assess their adherence to the management guidelines. Their monitoring reports were used to assess the performance of township and village levels and determine the performance related element of their pay. Each county government widely publicised the services to be provided and established a third-party (independent and objective) supervision group, including representatives of local political bodies, villagers, health managers and health programme technical staff, to ensure that the contracts were executed smoothly and fairly and that the reforms to basic public health and primary health care services were implemented appropriately.

The design of the intervention was based on the following specifications: (i) the government allocation would reach township and village facilities and the increase in funding for the provision of basic services would increase the incentives to provide public health and primary health care services; (ii) the publication of management guidelines and the linking of pay to the implementation of these guidelines would influence the performance of health service providers; (iii) county level facilities would take their monitoring role seriously and record any shortfalls, because they held the responsibilities for providing the technical assistance, training and designing technical standards in the health system; (iv) the publicity given to the new policy would encourage members of the community to put pressure on providers to deliver the services to which they are entitled and (v) the political supervision group would provide political support for the implementation of this policy.

### Sample selection

In July 2011 two counties (county 1 and county 2) were selected as intervention sites. They were similar in population, economic development, and in early progress with BPHS implementation (see Table 1). We also chose a county with similar characteristics as control. In the control county, the extra per capita was added to the usual block funding distributed to county, township and village health services, distributed in proportion to their total budgets. The control county was subject to the same goals, and received similar guidelines and targets as the intervention counties, but no direct support in the form of training was given; no accountability process was implemented, and thus no performance-based incentive/penalties; nor were the internal management structures altered to create collaboration between levels of care, autonomous decision making and decentralized management of the delivery of the service.

**Table 1:** Demographic characteristics at baseline

County	Population (10,000 people)	% of Age ≥65 years	GDP per capita (10,000 RMB)	Distance from City(Km)	No. of Township Centres	No. of Village Clinics
1	72.3	9.0	2.7	40	16	381
2	65.4	8.6	3.4	32	15	326
Control	60.4	8.9	2.9	55	15	343

Three townships were randomly selected from each of the three selected counties, which were stratified by net annual per capita income. Similar method was applied in the village selection procedure. The lists of villages were ranked by net annual per capita income, and one village were selected from each of the upper, middle and lower income group from each of the three townships. In total, nine villages in three townships in each county were selected.

We evaluated comparability of intervention and control villages, and compared registration and screening, referral and disease control and follow up by triangulating three data sources:

Population data for each village was obtained from population registers which are kept and updated by the local police station, and represent the authoritative listings of the population officially resident in that locality. These are frequently updated. In ten villages across all counties the accuracy of the population tallies was checked for those screened and registered for the BPHS in comparison with the official name lists of the residents, as registered at the local police station.

Patient lists were obtained from the local record system of the BPHS, which is held in each township hospital. The intervention counties have established a BPHS information management system covering counties, townships and villages based upon the electronic medical records established for each registered patient. All township hospitals and all but a few village clinics have fully functioning electronic medical records. The patients’ clinical file records and follow-up visits records were abstracted into a structured record by a specially trained research team visiting on site at clinics and hospitals, using name lists and identification numbers. Electronic data was transferred to USB key, viewed, and combined into a database. For paper records, data was input into an Excel spreadsheet, and entered into the database on the same day. For each of the selected villages, one list was prepared of all patients who, at recruitment to the BPHS, had been screened in the village as suspicious for diabetes on the basis of a random overnight fasting blood glucose test, and confirmed at the township hospital using clinical standards (overnight fasting glucose of ≥7.0 mmol/l). Another list was prepared of patients with a diagnosis of hypertension, who had been screened in the village on the basis of a single seated blood pressure measurement, and confirmed at the township hospital (using clinical criteria of systolic BP ≥ 140 mmHg and/or diastolic BP ≥ 90 mmHg).

### Data collection

Pre-/post-intervention data was collected for both intervention and control groups at the beginning (2011), the end of second year (2012) and the end of 2014. Baseline period was defined as January 1st, 2011 to June 30th, 2011. The BPHS intervention was initiated from July 1st, 2011, and ended as schedule at December 31st, 2014. January 1st, 2014 –December 31st, 2014 was defined as follow-up period in our analysis. In this study, the facility-level and patient-level data in 2014 were collected for evaluating the completeness of this project and health outcome achieved.

In this paper we evaluated effects of this intervention from five aspects, i) distribution of funds; ii) workload of health services providers; iii) payment to health workers; iv) number of patient referrals between different levels of health institutes; v) outputs of chronical disease management.

### Statistical analysis

Input data by using Epi Data (Version 3.1, software developed by “The Epi Data Association” Odense, Denmark), Univariate and bivariate analyses were conducted using PASW Statistics 18 (SPSS.Inc) run on Windows 7. Characteristics of intervention counties and control county at baseline were described by numbers and percentages. Comparative analysis was calculated between baseline and follow-up, and further compared between intervention and control groups. Estimates were presented with 95 % confidence level.

### Qualitative evaluation

Our evaluation team carried out in-depth interviews with more than 40 administrative staff and public health service providers in each county. We also reviewed routine management data from totally eight township health centres and 24 village clinics. Data including funds allocation, number of referral patients, number of registered chronical patients included in the primary health service package were collected at the beginning (2011), the end of second year (2012) and the end of 2014. Evaluations were focused on whether the logistics of the intervention are conducted accordingly. We examined the degree to which the intervention altered the understandings and behaviour of different stakeholders, including doctors, nurses, patients and health managers.

## Results

### Demographic characteristics at baseline

The intervention counties and the control county were similar in population and economic conditions. Demographic characteristics at baseline year are shown in Table 1.

### Allocation of funds

The county governments spent almost all the budgeted funds. The townships health centres received a high proportion of the contracted funds and paid a high proportion of sub-contracted funds to village clinics by the end of year 2014 (Table 2). These data suggest that the funds were allocated and spent as intended.

**Table 2:** Proportions of allowed amount from contracted government annual budget in the facilities with intervention (follow-up period, 2014)

County	Proportion of Allowed Amount Paid to Township Health Centres (%)	Proportion of Contracted Funds Paid to Village Clinics (%)
1	93.2	90.5
2	92.6	91.2

### Understandings of roles and provision of services

Prior to the intervention, the roles of township and village facilities were not clearly differentiated and they frequently competed for patients. After three years of implementation, the evaluation team found that the frequency of technical assistance between township health centres and village clinics had increased significantly (Table 3), Compared to the baseline period, average monthly training meeting and field supervision & guidance visits between township health centres and village clinics increased 1.0 meeting, 1.5 visits respectively, while the increases in control county were only 0.3 meeting and 0.3 visits, respectively. The township health facilities had increased the number of staff working on public health and primary health care services. They had required their more experienced clinical staff to spend one or two days a week in this work. The in-depth interviews found that most staff of township health centres believed that their workload had increased and their income increased at least more than 30 percent.

**Table 3:** Frequency of technical assistance between township health centres and village clinics

County	Average Monthly Training Meeting (No.)			Average Monthly Field Supervision & Guidance (No.)		
	Baseline	Follow-up	Difference	Baseline	Follow-up	Difference
1	1.2	2.2	1.0	1.1	2.6	1.5
2	1.1	2.0	0.9	1.0	2.4	1.4
Control	1.0	1.3	0.3	0.9	1.2	0.3

*Baseline definition: July- Dec 2011

*Follow-up definition: Jan- Dec 2014

The allocation of the performance-related payment was decided based on how well the service targets achieved in each health facilities. One indicator for a facility of good service quality was whether it received over 90 percent of their optimal performance-related pay. The proportion of township health centres achieving this indicator was 94.4 and 91.7 %, respectively in county 1 and 2. The proportion of villages reaching this indicator was 95.3 and 93.9 percent, respectively (Table 4). Health workers received 93.8 % of their optimal performance-related payment at the end of study in general. Some reasons for not achieving these targets were the heavy workload associated with the creation of new patient files for a large number of people and the problems that health workers experienced in learning their roles and responsibilities for new programmes such as diabetes, hypertension and serious mental illness. This lack of knowledge meant that they made errors in patient management.

**Table 4:** Proportion of health workers achieving performance goal at township level and village level

County	Township Health Centres (%)			Village Clinics (%)		
	Baseline	Follow-up	Difference	Baseline	Follow-up	Difference
1	88.2	94.4	6.2	90.5	95.3	4.8
2	84.1	91.7	7.6	82.8	93.9	11.1

**Fig. 1:**
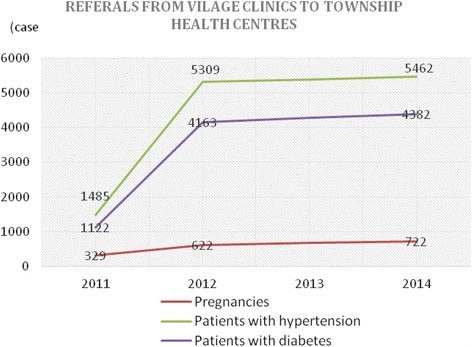
Number of patient referrals from village clinics to township health centres

**Fig. 2:**
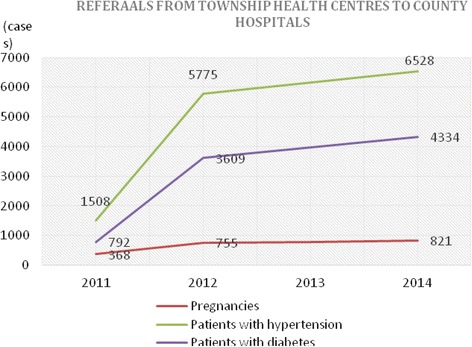
Number of patient referrals from township health centres to county hospitals

There were dramatic and continuous increases in the number of patients referred from village to township and from township to county levels for pregnancies (*n* = 8,997), patients with hypertension (*n* = 6,802) and patients with diabetes (*n* = 846) (Figs. 1 and 2). The details are presented in Figs. 1 and 2. Based on the evidence that the population of townships remained constant across study period, we provide the size of referrals in absolute value.

### Management for quality

The county monitoring teams visited township hospitals every three months, according to a contract the members signed with the county health bureau. The township hospitals organised supervision and guidance visits to villages at least once a month. The purpose of the visits was to identify problems and suggest actions. Hypertension management and diabetes management were selected as evaluation measures. The management follow up and control rate of patients with hypertension and diabetes after the intervention were statistically different between intervention counties and the control county. In intervention counties, there were 42.0 and 34.3 % increase of regular follow-up visit rate for diabetes and hypertension, respectively. Additionally, the system of health records which was established at the same time, successfully covered 96 % of the rural families. (Tables 5 and 6).

**Table 5:** Comparison of chronic disease management among patients with hypertension between intervention counties and control county in 2014 (follow-up period)

Indicators	County 1 (*N* = 176)			County 2 (*N* = 180)			Control County (*N* = 181)	
	n	%	*p*-value*	n	%	*p*-value*	n	%
Registration of hypertension patients	170	96.6	*p* < 0.001	155	86.1	*p* < 0.001	158	87.3
Regular follow-up visit rate of hypertension patients (%, n)	114	64.8	*p* < 0.001	124	68.9	*p* < 0.001	59	32.6
BP control rate of population under management (%)	62	35.2	*p* < 0.001	49	27.2	*p* < 0.001	12	6.6
Rate of referral and upper-level doctor’s service accepted by hypertension patients (%)	64	36.4	*p* < 0.001	56	31.1	*p* < 0.001	30	16.6

*p-value: Intervention county compared with control county by Chi-square test

**Table 6:** Comparison of chronic disease management among patients with diabetes mellitus between intervention counties and Control County

County	County 1 (*N* = 181)			County 2 (*N* = 153)			Control County (*N* = 181)	
	n	%	*p*-value*	n	%	*p*-value*	n	%
Registration of diabetes patients (%)	165	91.2	*p* ≤ 0.001	122	79.7	*p* ≤ 0.001	71	39.2
Regular follow-up visit rate of diabetes patients (%)	117	64.6	*p* ≤ 0.001	92	60.1	*p* ≤ 0.001	37	20.4
Blood glucose control rate of population under management (%)	56	30.9	*p* ≤ 0.001	46	30.1	*p* ≤ 0.001	24	13.3
Rate of referral and upper-level doctor’s service accepted by diabetes patients (%)	58	32	*p* ≤ 0.001	54	35.3	*p* ≤ 0.001	24	13.3

*p-value: Intervention county compared with control county by Chi-square test

## Discussion

Although many countries may need to allocate more resources to health services, global experience suggests that merely increasing existing block grants to service providers is unlikely to achieve the desired effects on service delivery, equity or effectiveness; changes to the mode of funding and of organization may both be needed to reach these goals [17–19]. The evaluation study found evidence that the counties had implemented the intervention as planned. They earmarked the agreed 20–35 RMB per capita for the basic package of services, signed service contracts and paid a high proportion of the budgeted amounts to township and village facilities. There were many intended effects. For example, the township health centres required senior clinicians to allocate time to public health and primary health care services and village doctors allocated a lot of time to these services. This study found substantial increases in regular follow-up visit rates for hypertension and diabetes. Intervention counties had created appropriate monitoring teams, although there was insufficient evidence on the contents of their monitoring visits and the degree to which they influenced performance.

This intervention demonstrates the usefulness of a performance-oriented approach that takes into account many influences on the performance of health service providers. This intervention focused on the provision of management guidelines, on new forms of payment on the basis of workload and achievement of service delivery targets and on informing the population about their new entitlements. Many pilot interventions succeed because of enthusiasm about something new and the attraction of financial rewards, but the initial gains are not always sustained as stakeholders adapt to the new pattern of incentives [9, 20]. Our study has the implications as follows: in order to achieve a sustainable change, it is important to: (i) ensure that health service providers benefit by delivering good quality services, (ii) establish effective monitoring arrangements to ensure that good performers are identified and (iii) create new public expectations about the services to which they are entitled. One factor that suggests that this can be achieved is the similarity between this intervention bears and successful to reduce maternal mortality [21]. Government officials made highly public statements in support of institutional delivery and contributed to a change in expectations about delivery. The county maternal and child health centres made regular monitoring visits to township hospitals to assess their performance in organising ante-natal care, institutional deliveries and post-natal care and their findings were taken into account in the overall performance assessment of the health centre directors. The obstetrics departments of the township hospitals experienced large increases in patient volume. It was difficult to interpret the rise in referrals of pregnancies from village to township levels because in 2011, it was already government policy for all deliveries to take place in an institution at township level or higher [22, 23]. The reported increase in referrals may simply be an artefact of the more formal management system. The referral from township to county levels may reflect a greater willingness to send patients to higher level facilities. The very large increase in the number of patients referred for treatment of hypertension and diabetes suggests that the township hospitals may experience a similar increase as a result of the county-wide screening effort [24]. The present study did not assess the full mix of treatment that people screened as having hypertension or diabetes received, or the full cost of treating them (both to themselves and to the rural health insurance scheme). Future evaluations of the impact of this intervention will have to assess trends in the cost of managing hypertension and diabetes.

One important factor in the success of the intervention was the capacity and commitment of the county government to allocate the required level of resources, monitor performance and enforce the contracts with health facilities. This illustrates the need for a strong government to engage effectively with health markets. Where government does not have this capacity it would be very difficult to make this kind of intervention work. It is important to keep this in mind in assessing the degree to which this experience is relevant elsewhere in China and in other countries.

The evaluation team identified some issues or study limitations that need attention as the intervention matures. First, the difficulty that many health workers had in newly introduced programmes to manage hypertension, diabetes and mental illness so training on these programmes should be organised in the future. Second, because major effort that had been made to create computerised patient records, more work will be needed to determine how these records should be used in managing patients and a monitoring the performance of the health system. Also, more attention should be given to the contents and impact of the monitoring visits.

## Conclusion

This combination intervention of performance-related contracts with health facilities, routine monitoring of performance and efforts to increase public awareness about the services demonstrates its effectiveness on improving the performance of health service providers. It is important for achieving a sustainable change in the piloted counties into a more efficient health system, which successfully engages a strong government role with health reform.
